# Supplementary material for: Paracetamol Intake and Hematologic Malignancies: A Meta-Analysis of Observational Studies
Source: J Clin Med. 2021 May 30;10(11):2429. doi: 10.3390/jcm10112429 (PMC8198062; doi:10.3390/jcm10112429)
Supplement: Supplementary file 1 [file jcm-10-02429-s001.zip › jcm-1182830-supplementary.pdf]

Paracetamol intake and hematologic malignancies: A meta-analysis of observational studies.

Jesús Prego-Domínguez [1] and Bahi Takkouche [1,2,*]

[1] Department of Preventive Medicine, University of Santiago de Compostela, 15782 Santiago de Compostela, Spain; susofis@gmail.com (J.P.D.); bahi.takkouche@usc.es (B.T.)
[2] Centro de Investigación Biomédica en Red de Epidemiología y Salud Pública (CIBER-ESP), 28029 Madrid, Spain
* Correspondence: bahi.takkouche@usc.es; (B.T.) Tel.: +34-881-812268; Fax: +34-981-572282

Table S1. Quality assessment of the studies included in the meta-analysis.

			CRITERIA[#] / SCORE[*]						
**Author**	**Year**	**Design**	**1**	**2**	**3**	**4**	**5**	**Total score**	
Friedman et al.	1981	Case-control	0	0	0	0	0	**0**	High risk
Kato et al.	2002	Case-control	0	1	1	0	1	**3**	
Friis et al.	2002	Cohorts	1	1	1	0	0	**3**	
Baker et al.	2005	Case-control	0	1	0	1	1	**3**	
Weiss et al.	2006	Case-control	0	1	0	1	1	**3**	
Moysich et al.	2007	Case-control	0	1	0	1	1	**3**	
Becker et al.	2009	Case-control	0	1	0	0	0	**1**	
Ross et al.	2011	Case-control	0	1	1	0	1	**3**	
Lipworth et al.	2003	Cohorts	1	1	1	0	1	**4**	Low risk
Chang et al.	2004	Case-control	0	1	1	1	1	**4**	
Walter et al.	2011	Cohorts	1	1	0	1	1	**4**	

Scoring criteria: 1) Participation in both groups> 80%; 2) Incident cases (case-control) / Changes of exposure accurately measured (cohorts); 3) Population controls (case-control) / loss of follow-up< 20% (cohorts); 4) Adjustment for sex, age and smoking or the distribution of these factors was similar between index and comparison group; 5) Duration of exposure accurately measured. * **Scoring method:** 0= no or not given, 1=yes; Score≥ 4: low risk of bias, score<4: high risk of bias.
